# Mycotoxigenic Fungi and Mycotoxins in Agricultural Crop Commodities in the Philippines: A Review

**DOI:** 10.3390/foods8070249

**Published:** 2019-07-08

**Authors:** Mark Angelo O. Balendres, Petr Karlovsky, Christian Joseph R. Cumagun

**Affiliations:** 1 Institute of Plant Breeding, College of Agriculture and Food Science, University of the Philippines Los Baños, College, Laguna 4031, Philippines; 2 Molecular Phytopathology and Mycotoxin Research, University of Göttingen, Grisebachstrasse 6, 37077 Göttingen, Germany; 3 Institute of Weed Science, Entomology and Plant Pathology, University of the Philippines Los Baños, College, Laguna 4031, Philippines

**Keywords:** *Aspergillus* sp., *Fusarium* sp., aflatoxin, fumonisin, ochratoxin

## Abstract

The tropical, warm, and humid conditions that are favorable to the growth and development of mycotoxigenic fungi put the Philippines at a high risk of mycotoxin contamination. To date, seven mycotoxigenic *Aspergillus* species, four *Fusarium* species, and one *Penicillium* species have been isolated from various agricultural crop commodities in the country. There are five mycotoxin groups (aflatoxin, fumonisin, ochratoxin, nivalenol, and zearalenone) that have been detected in both the raw form and the by-products of major crops grown in the country. Since the first scientific report of aflatoxin contamination in the Philippines in 1972, new information has been generated on mycotoxins and mycotoxigenic fungi, but little has been known of other mycotoxins until the last two decades. Further, despite the increase in the understanding of mycotoxigenic fungi and mycotoxins in the country, very limited knowledge exists on practices and measures that control both the fungi and the toxins. This paper reviews the current literature on mycotoxigenic fungi and mycotoxins in the Philippines with emphasis on the last two decades and on other mycotoxins.

## 1. Introduction

The United Nation Population Division (2017) [1] projects a global population of 9.8 billion people by 2050. Some scientists have argued that the earth’s resources, however, can only support 10 billion people. Assuming that this scenario is inevitable, there could be a rapid depletion of resources that would result in limited water availability and shortage of food supply. Agriculture and crop biotechnology’s solution is to increase food production by improving crop varieties through the development of drought-tolerant, high-yielding, and pest and disease-resistant crops. Another solution is to reduce crop yield loss caused by biotic and abiotic factors that are already limiting the current food supply. 

In the context of food security and safety, perhaps the most important pathogens of global significance are mycotoxigenic fungi [2,3]. This group of fungi can reduce the quality and quantity of marketable produce by damaging commodities, e.g., corn, rice, and peanuts, while producing mycotoxins metabolites that can be carcinogenic in both damaged and apparently healthy products or commodities. The latter is of grave concern because mycotoxins require analytical facilities or expensive kits for detection. Mycotoxigenic fungi and mycotoxins are a major concern worldwide, including the Asia Pacific region (e.g., Japan, Korea, Taiwan, the Philippines, Malaysia, Thailand, Indonesia, etc.) due to the presence of environmental and storage conditions that favor the growth and development of mycotoxigenic fungi [4]. Rice, for instance, is produced and consumed primarily in Asia and is very susceptible to mycotoxigenic fungi and mycotoxin contamination during storage. Developing countries are the most vulnerable to problems associated with mycotoxigenic fungi and mycotoxins [5], particularly with smallholder farmers during postharvest operations [6]. For instance, in Thailand, Indonesia, and the Philippines the total annual social cost of aflatoxin contamination in corn was estimated at 319 million AUS$ in 1991 [2,7,8]. Deaths of children in the Philippines due to lung infection has been associated to aflatoxin exposure [9]. 

In the Philippines, mycotoxigenic fungi significantly contribute to the decline in the quality and quantity of agricultural commodities. For instance, contamination of rice with mycotoxigenic *Fusarium fijikuroi* has been reported to cause seedling stunting and elongation known as bakanae disease [10]. Mycotoxigenic fungi are also common in staple crops (rice and corn) in the country (Table 1 and Table 2). Most *F. fijikuroi* isolates from rice are potential fumonisin producers [10] and isolates of *Fusarium verticillioides* that cause root, stalk, and ear rots in corn from Luzon, the Philippines are mainly fumonisin producers [11]. According to Salvacion et al. [12], most parts of the country are at a high risk of fumonisin contamination in both current and projected climate change scenarios. 

Mycotoxigenic fungi have also been found to contaminate the raw form and the by-products of cereals, grains, and non-cereal commodities (Table 2). Many toxins associated with these fungi have exceeded the maximum limits (20 µg kg^−1^) set by relevant food safety regulators (e.g., [21]. In a similar way to fumonisins, aflatoxins are also frequently detected in corn and rice. On the other hand, Ochratoxin A (OTA) has frequently been associated with coffee beans [13]. Regulatory control over the levels of aflatoxin in the Philippines has been set at the maximum of 20 µg kg^−1^ (pbb) for products consumed as human food [27] and produced for export [28]. These regulations complement those set in the CODEX standards and guidelines pertaining to food safety and the reduction of mycotoxin contamination, by the Food and Agriculture Organization (FAO), and the World Health Organization (WHO). However, the implementation of such regulation is not being followed because of the ignorance of most farmers and traders concerning aflatoxin contamination in corn, along with a lack of monitoring activities and the infrastructure to carry out aflatoxin testing [29]. The study recommends aggressive monitoring activities, more awareness campaigns, and the provision of aflatoxin testing kits in regional laboratories. 

To date, despite the increase in knowledge, mycotoxigenic fungi and their associated mycotoxins remain a significant threat to growers, consumers, and food regulators in the Philippines. This paper reviews the literature on the occurrences of mycotoxigenic fungi and mycotoxins in the Philippines, highlighting the increasing incidences of both in important agricultural crop commodities and the potential increase in the risk of fumonisin contamination. Quitco (1991) [30] and Arim (2000) [31] have made separate reviews on the status of mycotoxin research in the Philippines, with more attention on aflatoxin contamination. The current paper focuses on literature published within the last two decades (from 2000), with emphasis on aflatoxins, fumonisin, ochratoxins, and other minor but equally significant, mycotoxins. 

## 2. Mycotoxigenic Fungi

There are twelve mycotoxigenic fungi isolated from various crop commodities (Table 1) and their by-products. Corn, rice, peanuts, and copra grown in various regions of the country are commonly attacked by these mycotoxigenic fungi (Table 1). The largest group is *Aspergillus* sp., then *Fusarium* sp., and, finally, one species in the genus *Penicillium.*

### 2.1. Aspergillus Species

Up until 2000, only *Aspergillus flavus* and *A. niger* were the known mycotoxigenic *Aspergillus species,* and these were frequently associated with maize grains and groundnuts (Table 1)*;* however, research on coffee found five new species of *Aspergillus* that can produce mycotoxins. Alvindia and de Guzman (2016) isolated *A. carbonius*, *A. japonicus*, *A. ochraceous*, *A. niger*, and *A. westerdijkiae* in coffee beans and detected OTA for the first time in the Philippines. These species were isolated from the coffee-growing regions of Davao, Benguet, Ifugao, Abra, and Cavite [13]. Barcelo and Barcelo (2017) [22] similarly found *A. niger* and *A. ochraceus* in Arabica and Robusta coffee beans from the Cordillera Administrative Region (CAR) as well as the associated OTA. *A. niger* was frequently isolated in after-drying and roasted coffee beans [19]. These new findings bring a total of seven mycotoxigenic *Aspergillus* species contaminating major crop commodities in the country. Although no mycotoxin was detected, *A. flavus* contamination has also been reported for the first time in both fresh and dried banana chips [16], but the level of *A. flavus* colony forming units decreased as the drying period increased. It was also found that dust from copra, corn, and rice mills harbored *A. flavus* [18]. Rice by-products, e.g., bran, hull, brown, polished, etc., were found to be contaminated with *A. flavus* and *A. parasiticus* [18,32]. A high incidence (100%) of *A. flavus* was recorded in polished and brown rice [32]. Recently, *A. parasiticus* isolates were detected in soil samples from coconut fields in the Philippines and they all produced the four types of aflatoxins [23].

### 2.2. Fusarium Species

To date, there are four mycotoxigenic *Fusarium* species reported in the Philippines. Yamashita et al. [24] reported *Fusarium moniliforme* and *Fusarium proliferatum* contaminating corn grits. The first record of mycotoxigenic *Fusarium fujikuroi* in the Philippines was in rice collected from Laguna (Victoria) and Nueva Ecija (Muñoz) [10], both of which are rice-producing regions. Of the 32 *F. fujikuroi* isolates, 13 strains (41%) were FB1 producers [10]. The most frequently reported mycotoxigenic *Fusarium* species was *F. verticillioides*, which is commonly isolated in corn and is the predominant *Fusarium* species in the country [2]. Cumagun et al. [11] isolated *F. verticillioides* from corn cobs and detected isolates that are capable of producing fumonisin using polymerase chain reaction (PCR) specific primers. Magculia and Cumagun [33] isolated 49 *F. verticillioides* isolates from corn and 38 were capable of producing fumonisin. Pascual et al. [25] and Hussein et al. [26] also found a high incidence of *F. verticillioides* in corn kernels from the Philippines. Pascual et al. [25] did a comprehensive survey of *Fusarium* species associated with corn ear rot in the country and found that most of the *F. verticillioides* species are mycotoxigenic, regardless of their origin. Many of the regions that were surveyed by Pascual et al. [25], including the Southern Mindanao—a hotspot for corn ear rot disease—were also at very high risk for fumonisin contamination according to the prediction made by Salvacion et al. [12], particularly during the dry season [2]. Exacerbating the problem of fumonisin contamination is the occurrence of the sexual reproduction of *F. verticillioides* from the Philippines [34]. 

### 2.3. Penicillium Species

Alvindia and de Guzman [13] isolated twelve *Penicillium* sp. (*P. implicatum*, *P. montanense*, *P. purpurogenum*, *P. variabile*, *P. verruculosum*, *P. citrinum*, *P. corylophylum*, *P. decumbens*, *P. pelutatum*, *P. oxalicum*, *P. waksmanii*, and *P. digitatum*) from coffee beans grown in the highlands of Benguet and Ifugao. Of these twelve, only *P. verruculosum* was found to produce OTA in artificial media, as determined by high-performance liquid chromatography, at 7–12 µg kg^−1^ mycelium plug [13].

## 3. Mycotoxins

With OTA from coffee beans [13,22], there are five mycotoxins (fumonisin, aflatoxin, ochratoxin, nivalenol, and zearalenone) contaminating major crop commodities in the country (Table 2). Nivalenol and zearalenone were detected in corn, which were also positive for fumonisin and aflatoxin [24]. 

### 3.1. Aflatoxin

The four types of aflatoxin: B1, B2, G1, and G2 have been detected in copra, corn, coffee beans, peanuts, and rice (Table 2) and were detected (Table 3) at levels above the country limit (20 µg kg^−1^). Aflatoxin contamination in raw peanut kernels is a major problem during storage [35]. Aflatoxin B1 has been detected once in sorghum and soybean [14]. All four aflatoxin metabolites have also been detected simultaneously in rice bran and hull [17]. Dust in corn, copra, and rice mills was also found to contain aflatoxins. Aflatoxins in the Philippines are predominantly produced by *A. flavus,* which was found in many grain by-products, e.g., corn grits and polished rice [15,17]. Aflatoxins were also detected in corn grains infected with *A. parasiticus* [20]. Gonzales and Rafael [36] determined the aflatoxin contamination in corn in relation to moldy grain, moisture content, and grain damage in selected areas in Isabela. The hot spot for aflatoxin contamination was identified in Naguillan, Isabela. During the wet season, aflatoxin accumulation increased due to the high moisture content of the grain, but not during the dry season [36]. Animal feeds such as Madre de agua leaf meal were subjected to the same immunological technique. Aflatoxin was detected in the feeds in two provinces of Batangas and Quezon, but not in Los Baños, Laguna [37]. 

### 3.2. Fumonisin 

Fumonisin B1, B2, and B3 have been detected in corn (Table 2) and FB1 has been detected in rice [10]. *F. moniliforme, F. fijikuroi, F. proliferatum,* and *F. verticillioides* were responsible for FB1 contamination, with *F. fijikuroi* only found to colonize rice. Cumagun et al. [11] detected FB1, FB2, and FB3 in corn cobs contaminated with *F. verticillioides.* Fumonisin was also detected in corn isoline and Bt hybrids [39]. According to dela Campa et al. [39], the environmental conditions during silking are the most important factor for fumonisin accumulation. Using FumoniTest™ injected into a high-performance liquid chromatography (HPLC) system, the highest level of fumonisin for corn was found in Jones, with a mean value of 3.4 mg kg^−1^ during the wet season and Tumauini, with a mean value of 0.75 mg kg^−1^ during the dry season. All samples were contaminated with fumonisin, but only two percent exceeded the tolerable level of 5 mg kg^−1^ [42]. 

### 3.3. Ochratoxin 

Coffee bean contamination by OTA has recently been reported by several workers (Table 2). OTA was produced by five *Aspergillus* species, which are all first reports of mycotoxigenic fungi on coffee beans in the country. When grown in culture media, seventy percent of *A. ochraceus* isolates produced OTA of 16238 ng g^−1^, while 40% of *A. westerdijkiae* produced 36561 ng g^−1^. About 17% of *A. niger* produced OTA of 18439 ng g^−1^, 10% of *A. japonicus* of 174 ng g^−1^, and 21.21% of *A. carbonarius* yielded OTA of 1900 ng g^−1^. All these values were at the maximum levels. *A. niger* and *A. ochraceus* were frequently associated with OTA contamination [13,22]. OTA was more commonly detected in Robusta coffee (37%) than in Arabica coffee (26%) [40]. The highest level of OTA found in the dried whole cherries of Arabica was 97 µg kg^−1^, while for Robusta the highest level of 120 µg kg^−1^ was found [40]. The highest percentage of mycotoxigenic contamination was 98.4% [40].

### 3.4. Nivalenol and Zearalenone

Yamashita et al. [24] detected nivalenol and zearalenone in corn samples that were also positive to fumonisin and aflatoxin. Out of 208 maize samples from 18 provinces collected recently in the Philippines, 8.2% were positive for zearalenone, which is the first report of zearalenone in the country [43]. 

### 3.5. Cyclopiazonic Acid

Several species of *Penicillium* and *Aspergillus* produce mycotoxin cyclopiazonic acid. In the Philippines, cyclopiazonic acid was monitored in a single published report. Hayashi and Yoshizawa [44] reported cyclopiazonic acid in one of six corn samples from the Philippines that was intended for human consumption. 

## 4. National Standards

The Philippines National Standards (PNS) for the prevention and reduction of aflatoxins have been prepared by the Bureau of Agriculture and Fisheries Standards. Only three standards specific to aflatoxin exist, in addition to other related standards, e.g., the Code of Hygienic Practice for Processing and Handling of Corn Grits (PNS/BAFS 142:2015) and the Code of Good Agricultural Practices for Rice (PNS BAFPS 141:2014). The existing standards are the PNS on the Code of Practice for the Prevention and Reduction of Aflatoxin Contamination in Peanuts (PNS/BAFS 174:2015), in Corn (PNS/BAFPS 27:2008), and in Tree Nuts (PNS/BAFS 173:2015). Interestingly, no scientific paper has yet reported the occurrence of aflatoxin in tree nuts in the Philippines. Nevertheless, the code (PNS/BAFS 173:2015) has set a maximum allowable level of 10 ppb aflatoxin for all tree nuts, both ready-to-eat or those for further processing. Evidently, aflatoxin (and other mycotoxins) contamination in major crop commodities (Table 2) is mostly above the maximum limit allowed (20 µg kg^−1^ or pbb). These data indicate that while national standards exist, actions that reduce contamination have not been fully practiced, or practices may need to be modified. Additional storage facilities could also mitigate possible mycotoxin contamination. Comparison of aflatoxin and fumonisin B1 levels in maize samples in selected Association of South East Asian Nations (ASEAN) countries are shown in Table 4. These countries have more or less comparable tropical climatic conditions. 

## 5. Management

It is possible to remove infected kernels, beans, grains, etc. manually or by using sorting machines based on particle weight or optical techniques (for a review see [51]). Galvez et al. [35] successfully used manual sorting to remove groundnut grains with high levels of aflatoxin. In coffee beans, Barcelo and Barcelo [22] suggest that defective coffee needs to be removed during the initial post-harvest stage up to the drying stage. Recent technology that makes use of non-aflatoxigenic *A. flavus* to manage aflatoxins in peanuts in the Philippines, funded by the United States Agency for International Development (USAID) and in collaboration with the University of Georgia, Luis [52], identified four fungal strains of *A. flavus* that lacked the entire aflatoxin biosynthetic gene cluster and five strains with partial defects in the cluster. These nine atoxigenic strains are candidates for a biological control agent product in the Philippines. 

## 6. Conclusions

Mycotoxigenic fungi are pathogens of global importance. They damage the quality of agricultural crop commodities and impact negatively on food safety. In the Philippines, *Aspergillus, Fusarium,* and *Penicillium* species have been detected in crop by-products and were found to contaminate major crop commodities. The five major mycotoxin groups detected were aflatoxin, fumonisin, ochratoxin, nivalenol and zearalenone. Despite the knowledge gained in the last two decades, little information exists on control measures that would mitigate the effect of these mycotoxigenic fungi and mycotoxins in the country. Nevertheless, the increase of scholarly output indicates a growing interest by researchers on these fungi. Research outputs in the future could assist policy-makers in developing or improving standards and policies. 

## Figures and Tables

**Table 1:** Mycotoxigenic fungi isolated from agricultural crop commodities in the Philippines.

Species	Crop	Reference
*A. carbonarius*	Coffee	[13]
*A. flavus*	Banana, coffee, copra, corn, gabi, peanut, rice, sorghum, and soybean	[14,15,16,17,18,19,20,21]
*A. japonicus*	Coffee	[13]
*A. niger*	Coffee	[13,19,21]
*A. ochraceus*	Coffee	[13,19,22]
*A. parasiticus*	Rice and soil samples from coconut plantations	[17,18,20,23]
*A. westerdijkiae*	Coffee	[13]
*F. fijikuroi*	Rice	[10]
*F. moniliforme*	Corn	[24]
*F. proliferatum*	Corn and coffee	[19,24]
*F. verticillioides*	Corn	[11,25,26]
*P. verruculosum*	Coffee	[13]

**Table 2:** Mycotoxins, and their associated mycotoxigenic fungi, detected in agricultural crop commodities in the Philippines.

Toxin	Metabolite	Species	Crop	References
Aflatoxin	B1	*Aspergillus* sp. *A. flavus* *A. parasiticus*	Copra (meal and dust), corn (kernels, ground, feed, grits, cracked, whole, cornick, field corn, chips, puffs, popcorn, in cobs, germ, and dust), coffee (beans, beans in sack), peanuts (farmer’s stock, shelled, large segregated, and freshly dug), rice (brown, 2-year old in storage, dust, polished, bran, and hull), sorghum (in pile), soybean (in drying mat), and soil samples from coconut plantations	[14,15,17,18,21,23,24,38]
	B2	*Aspergillus* sp. *A. flavus* *A. parasiticus*	Copra (meal), corn (dust and in kernels), rice (dust, brown, polished, bran, and hull), and soil samples from coconut plantations	[17,18,23,24]
	G1	*Aspergillus* sp. *A. flavus* *A. parasiticus*	Corn (dust and in kernels), rice (dust, brown, polished, bran and hull), and soil samples from coconut plantations	[17,18,23,24]
	G2	*Aspergillus* sp. *A. flavus* *A. parasiticus*	Rice (bran and hull), corn (in kernels and ground), and soil samples from coconut plantations	[17,23,24]
	ni	*A. niger*	Corn (grains)	[21]
	ni	*A. parasiticus*	Corn	[20]
Fumonisin	B1	*Fusarium* sp. *F. fujikuroi* *F. verticillioides* *F. proliferatum*	Rice, corn (kernels, ground, grits, and in cobs)	[10,11,24,39]
	B2	*Fusarium* sp. *F. verticillioides F. proliferatum*	Corn (kernels, ground, grits, and in cobs)	[11,24,39]
	B3	*F. verticillioides*	Corn (in cobs)	[11]
Ochratoxin	A	*A. carbonarius* *A. japonicus* *A. niger* *A. ochraceus* *A. westerdijkiae* *P. verruculosum*	Coffee (beans)	[13,22,40]
Nivalenol	-	*Fusarium* sp.	Corn	[24]
Zearalenone	-	*Fusarium* sp.	Corn	[24]

ni, not indicated.

**Table 3:** Mycotoxin contamination levels in some agricultural crop commodities in the Philippines.

Toxin	Metabolite	Crop	Mycotoxin (Minimum–Maximum) Level (µg kg^−1^)	Above or below the Allowable Mycotoxin Limit (20 µg kg^−1^)	No. of Isolates/Samples	References
Aflatoxin	B1	Rice				
		Rough	17–368	above	45	[17]
		Brown	2–1274	above	125	[17]
		Polished	1–2546	above	61	[17]
		Hull	2–2641	above	34	[17]
		Bran	6–6173	above	14	[17]
		Dust	1.51–86.83	above	31	[18]
		Corn	1–430	above	50	[24]
		Whole	122 (mean), 369 (max)		4	[15]
		Grits	30 (mean), 80 (max)	above	10	[15]
		Cracked	10 (mean), 18 (max)	below	2	[15]
		Processed				
		Cornick	5 (mean), 12 (max)	below	17	[15]
		Chips	3 (mean), 8 (max)	below	31	[15]
		Puffs	1 (mean), 2 (max)	below	6	[15]
		Popcorn	2 (mean), 5 (max)	below	4	[15]
		Dust	1.01–30.08	slightly above	40	[18]
		Copra				
		Dust	5.21–21.38	slightly above	2	[18]
		Cake	0–2156	above	-	[31]
	B2	Rice				
		Rough	1–10	below	45	[17]
		Brown	1–20	below	125	[17]
		Polished	1–38	slightly above	61	[17]
		Hull	2–38	slightly above	34	[17]
		Bran	4–40	above	14	[17]
		Dust	0–4.78	below	31	[18]
		Corn	1–78	above	50	[24]
		Dust	0–2.63	below	40	[18]
		Copra				
		Dust	0.45–1.70	below	2	[18]
	G1	Rice				
		Rough	0–1	below	45	[17]
		Brown	0–1	below	125	[17]
		Polished	0	below	61	[17]
		Hull	1–6	below	34	[17]
		Bran	1–13	below	14	[17]
		Dust	1.01–30.08	slightly above	31	[18]
		Corn	40–78	above	50	[24]
		Dust	0–2.37	below	40	[18]
		Copra				
		Dust	0	below	2	[18]
	G2	Rice				
		Rough	0	below	45	[17]
		Brown	0	below	125	[17]
		Polished	0	below	61	[17]
		Hull	0–1	below	34	[17]
		Bran	0–1	below	14	[17]
		Corn	3–33	above	50	[24]
Fumonisin	B1	Corn	57–1820	above	50	[24]
		Corn	0–742	above	35	[11]
	B2	Corn	58–1210	above	50	[24]
		Corn	0–222	above	35	[11]
	B3	Corn	0–25	slightly above	35	[11]
Ochratoxin	A	Coffee				
		In drying yard	120.2 (mean)	-	122	[41]
		Roasted	4.8 (mean)	-	122	[41]
		In storage	46.7 (mean)	-	15	[13]
		Beans	8–36,561 (*Aspergillus* sp.)			
		Beans	7–12 (*Penicillium* sp.)	-	10	[13]
Nivalenol	-	Corn	18–102	-	50	[24]
Zearalenone	-	Corn	59–505	-	50	[24]

**Table 4:** Comparison of aflatoxin B1 and fumonisin B1 level in maize in selected Association of South East Asian Nations (ASEAN) countries with similar tropical climatic conditions.

Country	Aflatoxin B1 (µg kg^−1^)	Contaminated Samples (%)	Fumonisin B1 (µg kg^−1^)	Contaminated Samples (%)	Reference
Indonesia	5.6	66.7	226–1780	58.3	[24,45,46]
Malaysia	1.0–135	81.2	261–2420	100	[47]
Philippines	394.18–523,839.33	17.8	0.44–742	52.4	[11,43]
Thailand	73	85.7	63–18,800	89	[48,49]
Vietnam	20.5–110	26.4	5.6–89.8	23.5	[50]
